# A new modified Tsuge suture for flexor tendon repairs: the biomechanical analysis and clinical application

**DOI:** 10.1186/s13018-014-0136-x

**Published:** 2014-12-31

**Authors:** Jianghai Chen, Kun Wang, Foad Katirai, Zhenbing Chen

**Affiliations:** Department Hand Surgery, Wuhan Union Hospital, Tongji Medical College, Huazhong University of Science & Technology, Jiefang Ave. 1277, Wuhan, Hubei 430022 People’s Republic of China; Tongji Medical College, Huazhong University of Science & Technology, Hangkong Road 43, Wuhan, Hubei 430010 People’s Republic of China

**Keywords:** Flexor tendon, Repair, Modified Tsuge, Biomechanics, Clinical practice

## Abstract

**Purpose:**

This study is to develop a new suturing technique for flexor tendon repair by modifying the extant Tsuge repair techniques and to use biomechanical analysis to compare the new method with four established repair techniques and evaluate its clinical efficacy in the repair of 47 flexor tendons in 22 patients.

**Methods:**

The biomechanical analysis relied on 50 flexor digitorum profundus tendons harvested from fresh cadavers. The tendons were randomly divided into five groups, transected, and repaired by use of a 1. double-loop suture, 2. double modified locking Kessler, 3. four-strand Savage, 4. modified six-strand Savage, and 5. the new technique. The tensile force and breaking force of all repaired tendons were measured by static loading trials. For clinical application, 22 patients with acute flexor tendon injuries were treated with the new modified Tsuge suture and follow-up for more than 12 months.

**Results:**

While differences in the tensile force and breaking force in the modified Tsuge sutures and modified six-strand Savage sutures were not statistically significant, static loading trials showed the tensile force, in the form of a 2-mm gap formation, and the breaking force of the new modified Tsuge sutures were, statistically, both higher than the ones characteristic of double-loop sutures, double modified locking Kessler, and four-strand Savage sutures. After 12 months, restored functions were observed in all the patients during the postoperative 12 months. Total active motion (TAM) score demonstrated that more than 90% fingers were estimated as excellent or good.

**Conclusion:**

The new modified Tsuge sutures described here have evident higher tensile and breaking forces compared to other four-strand core suture techniques, suggesting, in turn, that this new technique is a good alternative for flexor tendon repairs in clinical applications.

## Introduction

Flexor tendon lacerations are common injuries treated by hand surgery. Even when such injuries are successfully repaired by specialists, however, postoperative complications, such as repair ruptures, gap formations, and adhesions can and do still arise in many patients [1,2]. Numerous studies have demonstrated the advantages of early active mobilization protocols in after-repair tendon healing processes, leading to a number of repair techniques being developed to increase the strength of repairs so that early active motion protocols can be implemented and functional outcomes of patients greatly improved [3–5].

Suturing techniques for flexor tendon repairs can be briefly categorized by the number of strands used for the core suture, such as two-strands, four-strands, and six-strands. Furthermore, they can be featured by properties, such as single- or double-stranded sutures. Double-stranded sutures (also called looped sutures) have been used in several repair techniques [6,7]. Although, in general, looped sutures have not yet been extensively marketed in many regions of Asia (for instance, China and India) and therefore their application is still obviously limited, among the looped sutures, Tsuge repairs and their modifications are accepted for their simplicity of manipulation in many countries. Notwithstanding this approbation, it has been noted by the authors and others that the strength of double-loop sutures, with four strands, modified from Tsuge repairs, still are, in some cases, inadequate for the high demands of early active rehabilitation [8].

The purpose of this study was to modify Tsuge sutures and to test their biomechanical properties both *in vitro* and *in vivo* applications. Single-stranded rather than double-stranded sutures, without many changes to the Tsuge repair configuration, were used to repair flexor tendons. The 2-mm gap formation tensile and ultimate breaking forces were then compared, *in vitro*, against the four established traditional repair techniques, followed, *in vivo*, by clinical evaluations of their efficacy. It was hypothesized that the new suture techniques, modified from Tsuge suturing techniques, would provide enough strength of repair to meet the requirements of early active mobilization protocols and would be a good alternative to Tsuge sutures when looped sutures were unavailable.

## Materials and methods

### Materials

The study was approved by the Committee on Clinical Research, Tongji Medical College, Huazhong University of Science and Technology. The biomechanical test used 50 flexor digitorum profundus tendons within zone III and zone IV, each 8 cm long, harvested from nine adult cadavers (seven males, two females) with an average of 67.4 years (range from 54 to 79 years). The cadavers were frozen for 2 to 9 weeks at −20°.

**Figure 1:**
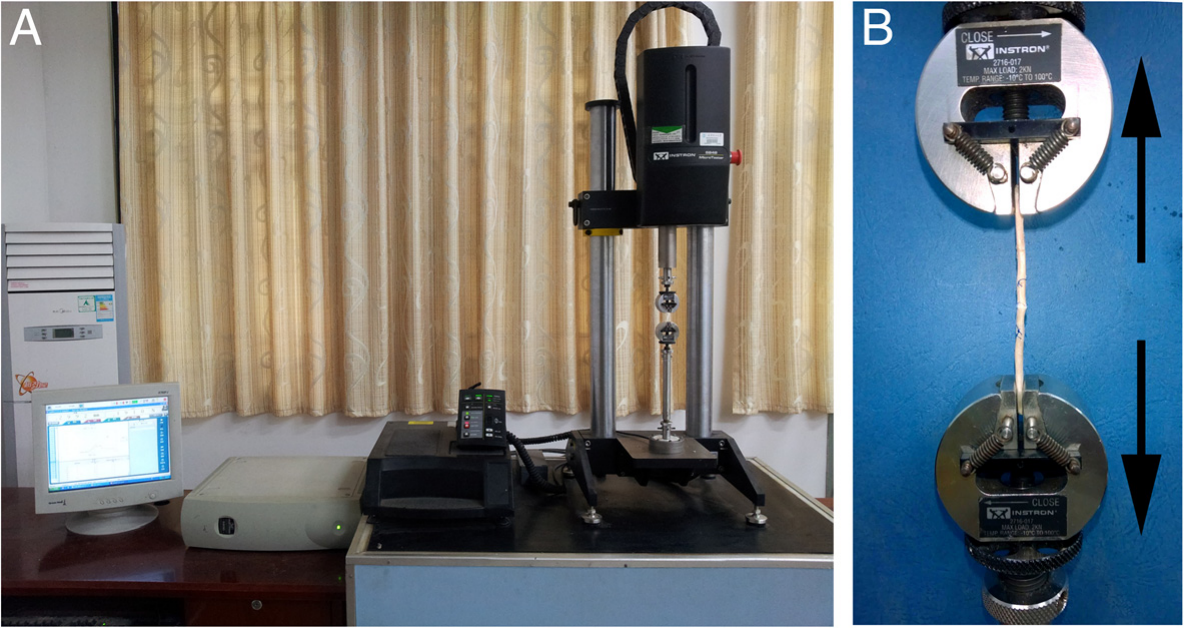
**The Instron Model 5848 MicroTester (A) and specimen attached to the tester (B).**

The biomechanical testing system, Instron Model 5848 MicroTester, purchased from Instron (in Norwood, MA), consisted of four components (Figure 1A):

- 5848 MicroTester frame,
- control tower,
- base tray,
- computer.

Specimen was attached to the Instron machine as in Figure 1B. Data was collected using an Instron Bluehill software.

### Suturing techniques

All repairs were performed by one single hand surgeon using the same type of instruments.

The new modified Tsuge suture was designed for and used in three steps using a single-stranded suture 4-0 monofilament polypropylene (Prolene, Ethicon, Somerville, NJ).

In the first step, the needle was laterally inserted into the proximal tendon end of the volar surface, within 1 cm from the intended repair site. The strand was then run longitudinally across the repair conjuncture and taken out 1 cm away from the repair site at the distal tendon end. With the needle passed transversely in the distal part, the strand was taken across the loop and the suture reinserted into the distal tendon end, crossing the repair site at the dorsal surface and existing from the proximal end dorsally, so that it could be reintroduced transversely to make a loop. A knot made in this site finished the first step.

**Figure 2:**
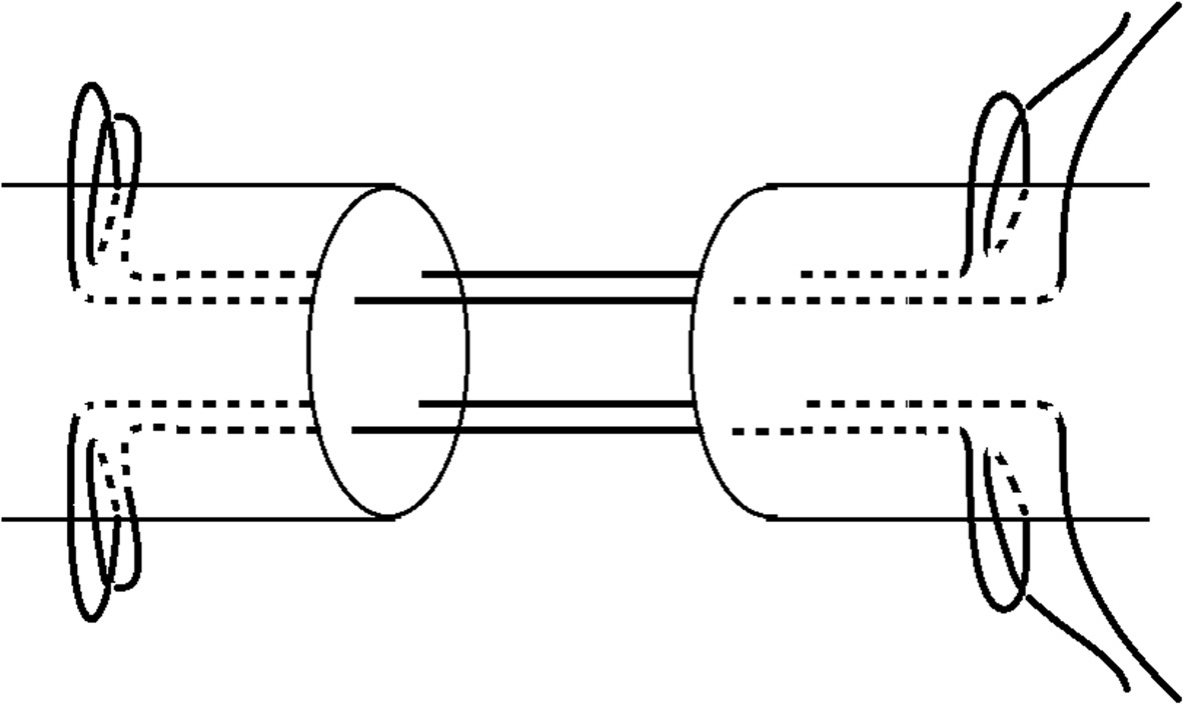
**The diagram of the new modified Tsuge suture.**

The second step involved the same procedure as the first step but exerted in the opposite side (Figure 2).

**Figure 3:**
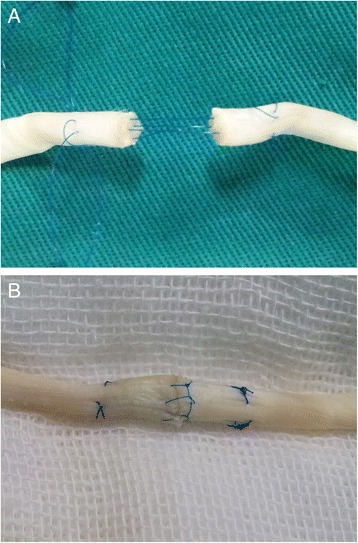
**The new modified Tsuge suture. (A)** After core-strand suture was carried out, four core strands which formed a cubic structure was observed. **(B)** The repair was finished with a peripheral running suture.

To complete the third step, the peripheral suture was placed as a running suture with 6-0 monofilament polypropylene (Figure 3).

The double-loop suture, double modified locking Kessler, four-strand Savage, and modified Savage (six-strand) were done using well-established procedures, which have been described by Viinikainen et al., without any modifications and completed by also adding a peripheral running suture [9].

### Biomechanical testing procedures

All tendons were properly fixed on clamps. A customary Hall effect sensor was positioned across the repair conjuncture to measure the tendon displacement. The repaired tendons were preloaded with an initial tension of 0.5 N. They were pulled at a speed of 0.2 mm/s. The 2-mm gap formation tension force and ultimate breaking force were measured and recorded.

Comparisons of tensile force for 2-mm gap formation and ultimate breaking force between the various repair groups were performed using a Tukey one-way analysis of variance with the level of significance set at *p* < 0.05.

### Clinical trials

From March to September 2011, 22 patients (17 males and 5 females), aged between 19 and 42 years (mean = 32 years), with 47 flexor tendon injuries were operated on at Wuhan Union Hospital, Wuhan, China. Of all the tendons, 11 had lacerations in zone II, 7 were injured in zone III, with the others injured in zone V. Among them, 7 were caused by sharp cuts, 13 were caused by saws, and 2 were crushing injuries. Nerve injuries in 12 of the patients were repaired at the same time. For postoperative rehabilitation, early active mobilization protocols described by Hung et al. were used [10]. Briefly, within the first two postoperative days, the hand was elevated and bound with compression dressings and a dorsal plaster slab. On the third day, the patients were asked to perform active mobilization of their hands under the supervision of a physical therapist. The fingers were actively but slowly and gently moved through 0°–50°. The number of movements was five times per section with three sections per day. The range of mobilization, the number of movements, and the times of section per day were evaluated by a therapist on a day-by-day basis and with an eye on the progress of the rehabilitation, increased gradually. Seven days after the operations, the patients were discharged and asked to accept physiotherapy, for another 14 days, and routine rehabilitation for 6 weeks.

### Functional estimation

The recovery of function for all patients was estimated by the use of the total active motion (TAM) scoring system (American Society for Surgery of the Hand). The evaluation was performed at 3, 6, 9, 12, and 24 weeks and 1 year postoperative.

## Results

### Biomechanical analysis

**Figure 4:**
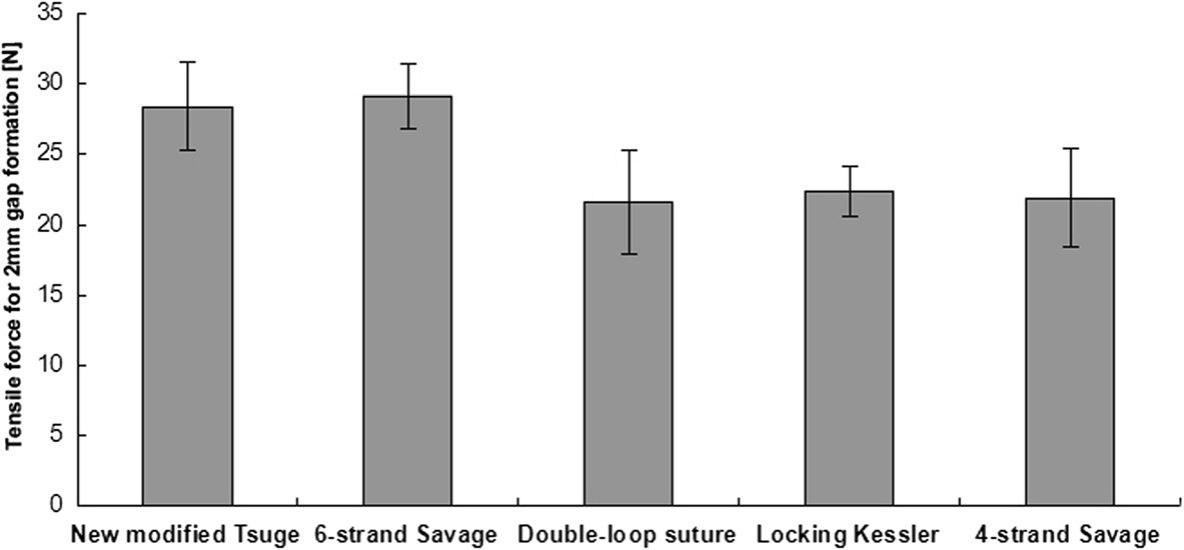
**Tensile force for 2-mm gap formation.** While it was comparable to tensile force from six-strand modified Savage repair, tensile force to generate a 2-mm gap was significantly higher in the new modified Tsuge suture when compared to other four-strand sutures (*p* < 0.05).

All tendons that were repaired with different techniques were subjected to static loading trials. Biomechanical testing showed that the new modified Tsuge sutures required almost the same tensile force to produce a 2-mm gap as the modified Savage (six-strand), while against the other four-stand core suture repairs, it presented significantly higher tensile gap forces. The mean force to generate a 2-mm gap in the new modified Tsuge was 28.4 ± 3.1 N. It was comparable to tensile force obtained from a modified Savage repair (29.1 ± 2.3 N) technique. Impressively, the force needed in the new modified Tsuge was significantly higher than that in double-loop sutures (21.6 ± 3.7 N), double modified locking Kessler sutures (22.4 ± 1.8 N), and four-strand Savage (21.9 ± 3.5 N) sutures (Figure 4).

**Figure 5:**
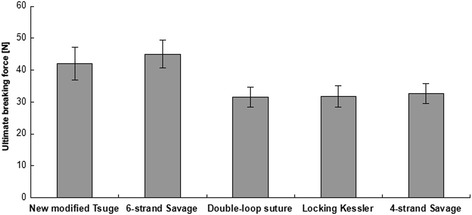
**Ultimate breaking force.** Ultimate breaking force was significantly increased in the new modified Tsuge suture compared with double-loop sutures, double modified locking Kessler, and four-strand Savage sutures (*p* < 0.05).

On the other hand, the breaking force of the new modified Tsuge suture was 42 ± 5.2 N, while for the modified Savage, it was 45 ± 4.3 N. With similar patterns for tensile gap forces, the ultimate tensile force was significantly stronger in the new modified Tsuge sutures than in double-loop sutures (31.5 ± 3.1 N), double modified locking Kessler (31.8 ± 3.4 N), and four-strand Savage (32.7 ± 3.1 N) sutures (Figure 5).

These *in vitro* results showed the new modified Tsuge to be a higher provider of repair strength during early active mobilization when compared with other four-strand core suture techniques.

### Clinical application

**Figure 6:**
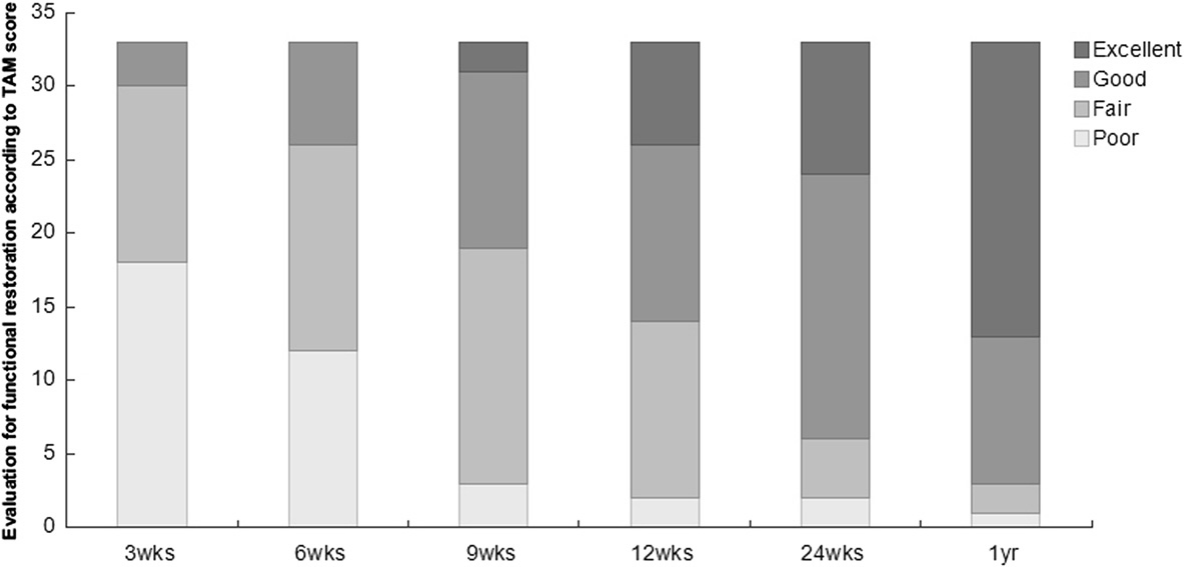
**Evaluation of functional restoration.** Functional restoration for all patients was estimated using the TAM score system at different time points.

All operations were performed by two experienced hand surgeons (J.C. and K.W.). Operation time for repairing tendons in each patient was varied from 6 to 75 min depending on the number of lacerated tendons. In average, the time of repairing for one tendon was 7 min. Postoperative rupture was not observed in any patient during follow-up. For all 22 patients (33 fingers, 47 flexor tendons), estimation of functional recovery using the TAM score system was exerted at different time points (3 weeks, 6 weeks, 9 weeks, 12 weeks, 24 weeks, and 1 year) and showed excellent and good score results for 30 fingers (91% in total) after 1 year postoperative. One year postoperative, three fingers were evaluated with fair or poor results. Among of them, one of the fingers from zone II injury group was measured as poor with the TAM score system. One finger with the zone II injury and another one with zone V injury were estimated as fair. The results revealed progressive range of movement restoration (Figure 6, (Table 1)).

**Table 1:** **TAM scores of all fingers in according to zones of transection**

	**Zone II**	**Zone III**	**Zone V**
Excellent	4	4	12
Good	5	1	4
Fair	1		1
Poor	1		

After 1 year postoperative, one of the fingers from zone II injury group was measured as poor with TAM score system. One finger with zone II injury and another one with zone V injury were estimated as fair.

## Discussion

Peritendinous adhesions have often proven to be a major problem for functional recovery of the hands [11,12]. Early active mobilization has been considered as one of important factors for successful functional restoration after flexor tendon injury [13]. In the present study, a new modified Tsuge suture was employed to repair tendon injuries. *In vitro* biomechanical analysis showed that the new technique could provide enough tensile strength to implement early active mobilization protocols. Furthermore, preliminary clinical trials proved that this suture can lead to satisfied functional outcomes with well-organized postoperative physiotherapy.

Postoperative repair strength for tendon injuries are mainly determined by the repair techniques employed, including material properties, knot security, and suture configuration [9]. Commonly, the suture techniques used for flexor tendon repair are composed of two parts, core sutures, and peripheral sutures. While peripheral sutures are similar among different repairs, the number of strands for the core suture differs with different techniques. Applications of two-strand repairs, widespread in clinical practice, are strong enough to exert early passive motion [14]. However, several studies have reported increased rupture rates for two-strand repairs after using early active mobilization protocols [15,16]. Therefore, it is not surprising that many multiple-strand sutures have been designed to meet clinical demands. Savage et al., for example, first described a six-strand core suture, while modified Kessler, modified Becker, and the triple-loop suture, etc. were reported as having been used to repair flexor tendon injuries with six- or eight-strand core sutures [17–22]. Nevertheless, while these techniques have proven to improve tensile force for gap formation and ultimate breaking force, their complex properties lead to several limitations in clinical practice. For instance, it took injured tendons markedly increase operating times due to the complicated configuration of six- or eight-strand core sutures. Higher gliding resistance also resulted from these repair methods. Balancing of loads has proven difficult to control using these techniques. Difficulties in the learning process may also have discouraged widespread acceptance of these repairs [23–26]. Not unexpectedly, many hand surgeons would like to see more well-balanced techniques in their practice.

**Figure 7:**
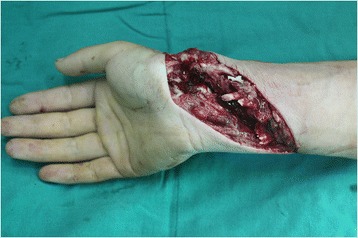
**A 26-year-old male was injured in zone V by sharp cut (broken glass).** All FDS and FDP tendons, FPL tendon, FCR tendon, and FCU tendon were disrupted. Laceration of radius artery and ulna artery were observed. The median and ulnar nerves were also injured.

**Figure 8:**
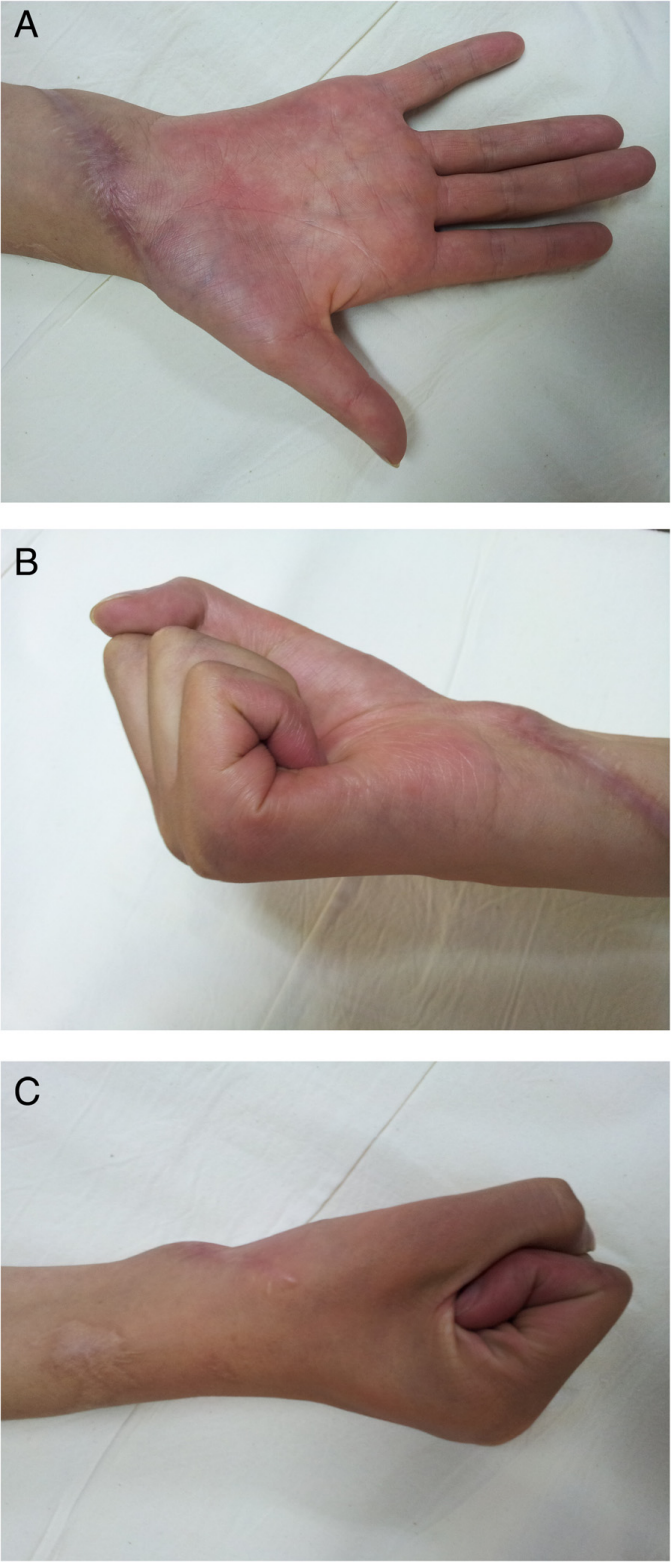
**Six months postoperative, the ultimate test in the form of flexion (B,C) and extension (A) of the injured hand proved to be excellent.**

Tsuge sutures are widely accepted on account of their simpler configurations. However, to date, the looped suture needed by this technique has not been well marketed in many countries (such as China, India, and most countries in Africa). The design of the new modified Tsuge suture, which can be implemented with single-stranded suture, was the focus of this paper. In this study, biomechanical testing showed new technique provided similar tensile force for 2-mm gap formation and ultimate breaking force to six-strand Savage suture and higher forces than other four-strand suture approaches. The clinical results demonstrated that this repair was strong enough to support early active mobilization protocols for obtaining satisfied functional recovery. This interesting result might be derived from two important factors from the new technique. The first one is that this new modified Tsuge suture produces a cubic core suture structure when repairing injured tendons. This configuration provides more balanced loads compared to other four-strand core sutures. The second one is that the lock configuration of longitudinal and transverse strands, instead of grasp loop, at points A, B, C, and D (Figure 2) contribute to enhanced forces as well. Therefore, the 2-mm gap formation and ultimate breaking force of the new modified Tsuge sutures were significantly higher than those of double-loop sutures and double modified locking Kesslers, four-strand Savage and comparable to the modified Savage with a six-strand core suture. Moreover, there are a few advantages for the new technique when compared to others. For instance, the operating time needed to use this technique is also less by our experience. In this study, surgeons spent around 7 min to repair one tendon in average. We spent around 75 min to repair 11 tendons in a 26-year-old male patient (Figures 7 and 8). Learning the technique was also an advantage. Twenty-eight resident doctors who were taught with the new technique all learned it within 30 min (date is not shown in here), showing how the new repair is easy to be learned even if surgeons have no prior experience with Tsuge sutures. In addition, the surgeons needed only one single-stranded 4-0 suture and one 6-0 suture to repair one tendon, saving substantial material costs for many patients, especially in multiple tendon injuries.

In conclusion, the new modified Tsuge technique is a simpler suture, providing stronger tensile force and breaking force. It is a good alternative when looped sutures are unavailable.

### Consent

Written informed consent was obtained from the patient for the publication of this report and any accompanying images.
